# Rescue of αB Crystallin (HSPB5) Mutants Associated Protein Aggregation by Co-Expression of HSPB5 Partners

**DOI:** 10.1371/journal.pone.0126761

**Published:** 2015-05-11

**Authors:** Rasha M. Hussein, Ivor J. Benjamin, Harm H. Kampinga

**Affiliations:** 1 Department of Cell Biology, University Medical Center Groningen, Groningen, The Netherlands; 2 Department of Biochemistry, Faculty of Pharmacy, Beni-Suef University, Salah Salem Street, 62511, Beni-Suef, Egypt; 3 Cardiovascular Center, Medical College of Wisconsin, Milwaukee, Wisconsin, United States of America; University of Colorado Denver School of Medicine, UNITED STATES

## Abstract

HSPB5 (also called αB-crystallin) is a ubiquitously expressed small heat shock protein. Mutations in HSPB5 have been found to cause cataract, but are also associated with a subgroup of myofibrillar myopathies. Cells expressing each of these HSPB5 mutants are characterized by the appearance of protein aggregates of primarily the mutant HSPB5. Like several members of the HSPB family, HSPB5 can form both homo-oligomeric and hetero-oligomeric complexes. Previous studies showed that co-expression of HSPB1 and HSPB8 can prevent the aggregation associated with the HSPB5 (R120G) mutant in cardiomyocytes and in transgenic mice. In this study, we systematically compared the effect of co-expression of each of the members of the human HSPB family (HSPB1-10) on the aggregation of three different HSPB5 mutants (R120G, 450 Δ A, 464 Δ CT). Of all members, co-expression of HSPB1, HSPB4 and HSPB5 itself, most effectively prevent the aggregation of these 3 HSPB5 mutants. HSPB6 and HSPB8 were also active but less, whilst the other 5 HSPB members were ineffective. Co-expression of Hsp70 did not reduce the aggregation of the HSPB5 mutants, suggesting that aggregate formation is most likely not related to a toxic gain of function of the mutants *per se*, but rather related to a loss of chaperone function of the oligomeric complexes containing the HSPB5 mutants (dominant negative effects). Our data suggest that the rescue of aggregation associated with the HSPB5 mutants is due to competitive incorporation of its partners into hetero-oligomers hereby negating the dominant negative effects of the mutant on the functioning of the hetero-oligomer.

## Introduction

HSPB5 (also called αB-crystallin) is a member of the ATP-independent HSPB family (small HSP) characterized by a conserved α-crystallin domain. The small HSP are thought to prevent protein aggregation and to assist in the folding of proteins in cooperation with the ATP-dependent HSP70 machinery or/and to promote the degradation of clients by ubiquitin proteasomal or autophagic lysosomal systems [1,2]. HSPB family members are known for their ability to form both homo-oligomeric and hetero-oligomeric complexes with other HSPBs. These complexes are dynamic, show high rates of subunits exchange, and suggested to play a key role in substrate recognition and chaperoning functions of the HSPBs [1,3].

HSPB5 is expressed at high levels in the lens but is also expressed in tissue-restricted manner in other non-lenticular tissues such as the heart, skeletal muscles and the brain [4]. In the lens, HSPB5 forms a hetero-oligomeric complex together with HSPB4 (αA-crystallin) in a 1:3 ratio forming the α-crystallin protein complex, which constitutes 50% of the total lens proteins. Besides being a major structural protein in the lens, α-crystallin protein maintains the transparency of the lens and prevents the cataract formation by suppressing the aggregation of the proteins in the eye lens such as β and γ crystallins [5,6]. HSPB5 and HSPB4 are believed to originate from a duplication of an ancestral α-crystallin gene and show 57% amino acid sequence homology [7]. In addition to the functions of HSPB5 in the eye lens, the biochemical activity of HSPB5 was also found to be important in other tissues where it modulates the angiogenesis, inflammation, and promotes the cytoskeleton stabilization, and seems to assist in protein degradation [8,9]. In vitro, HSPB5 can prevent the aggregation of several client proteins upon chemical denaturation such as denatured β and γ crystallin [10,11] or upon thermal denaturation such as heat denatured citrate synthase [12] and amyloid formation by amyloid beta (Aβ42) peptides [13] and beta 2 microglobulin [14].

Interestingly, 15 different mutations in HSPB5 gene were found to associate with a subgroup of the myofibrillar myopathies referred to as α-crystallinopathy [15] or chaperonopathies [16]. Although the clinical features of the myofibrillar myopathies are heterogonous, the pathological mechanism of these heterogonous diseases always starts with disintegration of the Z-disk of the myofibrils followed by accumulation of myofibrillar degradation products [17–19]. Expression of these HSPB5 mutants is found to be associated with formation of protein aggregates that sequester the mutants themselves. It remains unclear whether the aggregation is due to effects of the mutant (e.g., dominant negative) on the functionality of HSPB chaperone complexes or whether the mutants have gained a toxic function, or both. In case of the latter, promoting the overall chaperone activity of cells should rescue the phenotype, provided that the mutants are detected as misfolded clients. Likely, different mutants would adopt different folds and thus require different chaperones for handling. If acting through dominant negative effects, rescue maneuvers may be possible either by overexpression of wild type HSPB5 or other oligomeric partners of HSPB5 independent of the type of mutation.

In this study, we tested this hypothesis by focusing on three different HSPB5 mutants (R120G, 450ΔA, 464ΔCT). The missense mutation, HSPB5 (R120G) is transmitted by autosomal dominant inheritance and associated with both cataract and adult onset myopathy. When expressed in cells, the R120G mutant is associated with formation of amyloid-like oligomeric structures that are often found closely located to so-called aggresomes [20]. In vitro, the R120G mutation was found to impair the normal stability and oligomerization of HSPB5 protein through disrupting its ability to form dimers [21] and has decreased chaperoning activity [22,23]. Recombinant HSPB1 and HSPB8 could prevent aggregate formation mediated by HSPB5 (R120G) protein *in vitro*. In cultured cells, HSPB1 and HSPB8 overexpression prevented the aggregation of R120G [24,25] and cardiac-specific HSPB8 overexpression prevented the aggregation and the cardiomyopathy progression in R120G transgenic mice [26]. Like the R120G mutation, the 450ΔA mutant causes disease through dominantly inheritance but it is only associated with congenital posterior polar cataract with no myopathy detected in patients bearing this mutation [27]. The other truncating mutation (464 ΔCT) results from deletion of two nucleotides with a complete change of the C-terminal sequence after Gly154. It is associated with dominantly inherited adulthood muscle weakness, cervix and limb girdle myopathy and respiratory insufficiency [28]. In cells, HSPB1 could also rescue the aggregation of 464 ΔCT HSPB5 [29]. However, other HSPB members were not tested so far.

These results prompted us to systematically screen all HSPB family members (HSPB1-10) to discover which ones can prevent the aggregation of any of these 3 HSPB5 mutants (R120G, 450 ΔA, 464 ΔCT). Strikingly, we found that only those HSPB members that are known to form hetero-oligomeric complexes with HSPB5 are able to prevent the aggregation of HSPB5 mutants. Co-expression of Hsp70 did not reduce the aggregation of the HSPB5 mutants. Further, we found not only that HSPB4 interacted with both HSPB5 wild type and mutant equally well but that its capacity to prevent aggregation was unrelated to increased degradation of the HSPB5 mutants. Our findings thus suggest that rescuing of the aggregation associated with the expression of the HSPB5 mutants by overexpression of its binding partners negates the dominant negative effects of the mutant on the hetero-oligomeric chaperone complexes.

## Materials and Methods

### Plasmid construction

HSPB5 mutants used in this study: pCMV-myc-HSPB5 (R120G), pCMV-myc-HSPB5 (450 Δ A) and pCMV-myc-HSPB5 (464 Δ CT) as in reference [29]. PCMV-myc-HSPB5 (Wt) was produced by in vitro site directed mutagenesis from pCMV-myc-HSPB5 (R120G) plasmid using the primers: 5'- GGA TCC GGT ATT TCC TGT GGA ACT C-3' (Biolegio BV, The Netherlands). The small heat shock proteins plasmids FRT-TO-V5-(HSPB1-10) were described before in reference [30].

### Cell culture and transfections

Human embryonic kidney cells stably express tetracycline repressor (Flp–In T-REx HEK 293; Invitrogen, Carlsbad, CA, Catalog number: R780-07) and mouse myoblast C2C12 cells (ATCC, CRL-1772 Manassas, VA) kindly provided by Angelo Poletti, Milan, Italy were grown in Dulbecco’s Modified Eagle Medium (DMEM, GIBCO). The medium was supplemented with 10% Fetal calf serum (Greiner Bio-one, Long wood, FL, USA) plus 100 units /ml penicillin and 100mg/ml streptomycin (Invitrogen) and cells were grown at 37C° under a humidified atmosphere containing 5% CO_2_. Blasticidin (5 μg/ml, GIBCO, Invitrogen) was regularly added in the culture medium of Flp—In T-REx HEK 293 cells and tetracycline (1μg/ml, Sigma) was added to switch on the expression of FRT-TO-V5-(HSPB1-10) when needed. HEK293 cells were plated at 2 x10^5^ density into poly-L-lysine (0.001%, Sigma) coated wells for 24 h. before transient transfections. 0.5 μg of different HSPB5 mutants were transfected into HEK293 by PEI transfection reagent (1 μg/ μl, Polysciences). For C2C12 cell line, plating the cells and transfection was done at the same time by Lipofectamine 2000 (Invitrogen) according to [31]. Both cell lines were transiently transfected for 48 h before lysis.

### Cell fractionations and Western blotting

Cells were washed twice with ice-cold PBS and lysed into lysis buffer A (50 mM Tris–HCl, pH 7.4, 150 mM NaCl, 1 mM EDTA, 1% Triton X-100) supplemented with complete Protease inhibitor Cocktail (Roche Diagnostics, Germany). Cell lysates were centrifuged at 12000 rpm for 10 min at 4 C°. The supernatants were collected and used as soluble fractions while the pellet fractions were washed once with PBS and dissolved into SDS buffer (50 mM Tris–HCl, pH 6.8, 2% SDS, 100 mM DTT, 10% Glycerol). Pellet fractions were sonicated for 30 sec. by SONIFIER B-12. All samples were mixed with SDS sample buffer supplied with 5% 2- mercaptoethanol (Sigma) and boiled for 10 min. Samples were loaded onto 12.5% glycine SDS-PAGE gels followed by transfer into nitrocellulose membranes. Membranes were blocked with 5% dry milk in PBS with 0.1% Tween 20 (PBST) for 1 h at room temperature and incubated overnight at 4 C° with the following primary antibodies: Anti myc (1:2000 in PBST, MBL, Japan), anti V5 (1:5000 in PBST, Invitrogen), anti β-actin (1:1000 in PBST, Abcam). The next day, membranes were washed with PBST and incubated with anti-mouse HRP-conjugated secondary antibody (1:5000 in PBST, GE Healthcare) for 1 h at room temperature. Enhanced chemiluminscence was used for protein detection using ECL western blotting substrate kit (Thermoscientifc). Bands were visualized by exposure of the membranes to Amersham Hyperfilm ECL (GE Heath Care, UK).

### Immunofluorescence microscopy

One day before transfection, HEK293 cells were plated into 0.001% poly L-lysine coated cover slips. Cells were transiently transfected with 0.5 μg of different HSPB5 mutants and either 1.5 μg of HSPB4-V5 or FRTTO for 48 h. Cells were washed twice with PBS, fixed with 3.7% formaldehyde in PBS for 15 min and permealized with 0.2% Triton X-100 in PBS for another 15 min. Cells were blocked with 100 mM glycine for 10 min then with 3% BSA in PBST for 30 min. Cells were incubated with primary antibodies: mouse anti myc (1:200 in PBS,MBL Japan) and rabbit anti V5 (1:200 in PBS, Invitrogen) overnight at 4 C°. Coverslips were washed with PBS-T (3x 5 min) and incubated with anti-rabbit Alexa fluor 488 (1:250 in PBST, Invitrogen) and anti mouse Alexa Fluor 594 (1:250 in PBST, Invitrogen) for 1 h. at room temperature. Coverslips were washed again with PBS-T and incubated with 0.2 μg/ml 4,6-diamidino-2-phenylindole (DAPI) for 10 min to stain the nuclei. Coverslips were washed with PBS and mounted with Citifluor medium (Citifluor Ltd, London, UK). Images were obtained with Leica TCS SP8 confocal laser scanning microscope with HC PL APO CS2 63x/1,4 oil objective.

### Cycloheximide chase assay

HEK293 Cells were transfected with 0.5 μg of different HSPB5 mutants and either 1.5 μg of HSPB4-V5 or FRTTO for 24 h. Cells were treated with cycloheximide (50 μg/ml, Sigma-Aldrich) for 2, 4, 6, and 8 hours. Cells were washed with PBS before lysis into SDS buffer and sonication. SDS-PAGE and Western blotting were performed as mentioned above.

### Co-Immunoprecipitation

After cell transfection, cells were washed with ice-cold PBS and lysed in lysis buffer A (50 mM Tris–HCl, pH 7.4, 150 mM NaCl, 1 mM EDTA, 1% Triton X-100) + 1mM DTT (Dithiothreitol) + complete protease inhibitor cocktail (complete TM, Roche Diagnostics, Germany). For each reaction, A/G agarose beads (Protein A/G plus agarose, Santa Cruz Biotechnology) were incubated with rabbit anti V5 antibody (Invitrogen) in 20:1 ratio at 4 C° for 1 h with slow agitation (10 rpm) and washed once with lysis buffer before use. Cell lysates were centrifuged at 10000 rpm at 4 C° for 15 min, the supernatant was separated into a new tube and used as the input fraction. The input fraction was precleared first by incubating with A/G agarose beads at 4 C° for 1 h with slow agitation. Finally, the precleared input was incubated with anti V5-coated beads at 4 C° with slow agitation overnight. Input fractions were then centrifuged at 8000 rpm at 4 C° for 1 min and the resulting beads were washed 3–4 times with lysis buffer at the same centrifugal force. Proteins were dissolved in SDS sample buffer and boiled for 5 minutes. SDS-PAGE and western blotting were performed as mentioned before using mouse anti-myc and rabbit anti V5 antibodies to detect HSPB5 mutants and HSPB4 respectively.

### Data analysis

Data are expressed as mean ± SD of at least 3 independent experiments. Densitometry of western blot bands was performed using Image Studio Lite software, LI-COR, Biosciences, U.S.A.

## Results

HSPB5 mutants have been found to be associated with intracellular aggregates formation both in disease and when expressed ectopically in cells. Whether or not aggregate formation of the mutants themselves are associated with a gain-of-toxic mechanisms such as the poly glutamine proteins in CAG repeat expansion diseases [32] or due to a loss of function (LOF) is yet unclear.

**Fig 1:**
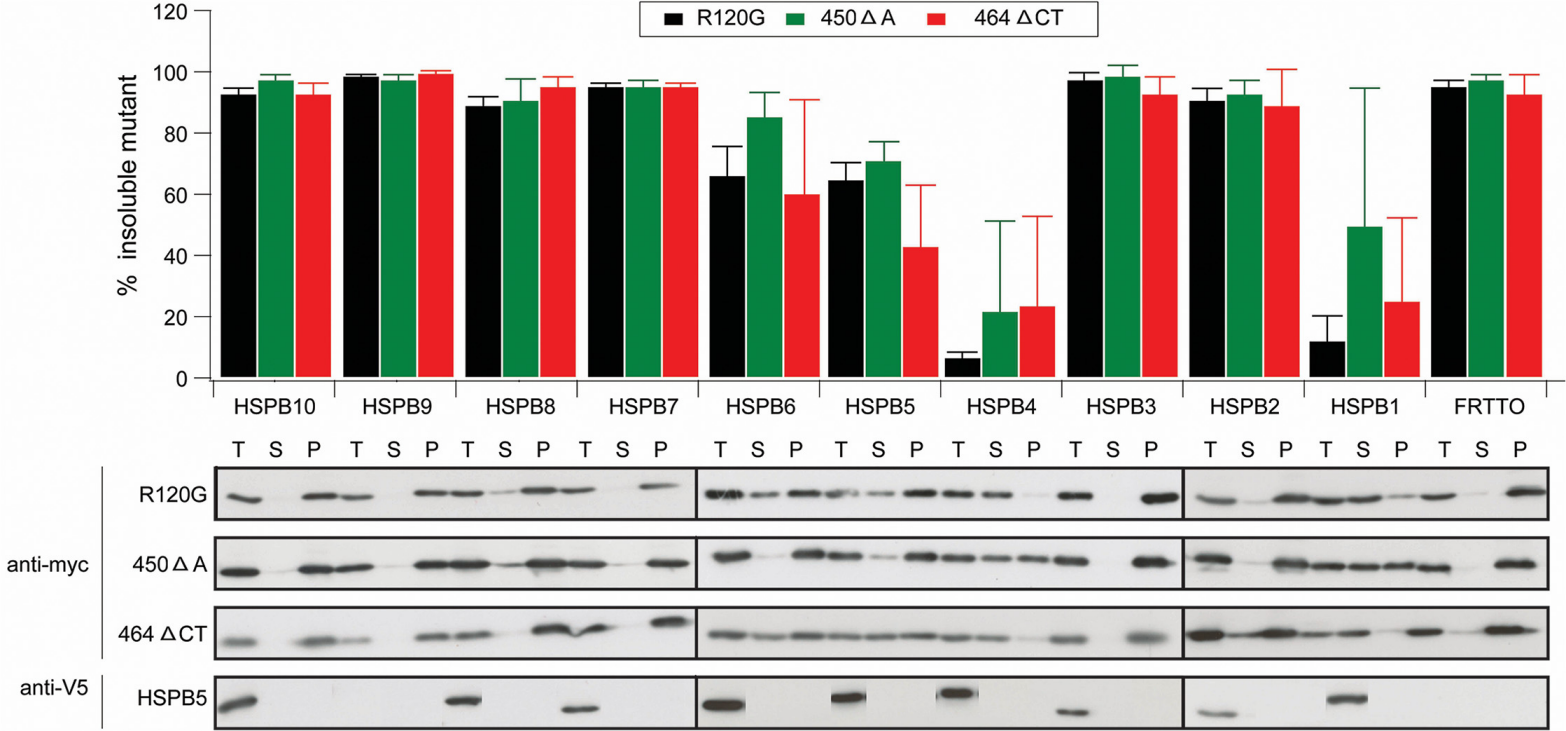
Effect of the 10 human HSPB family members on the aggregation of HSPB5 mutants. HEK293 cells were co-transfected with 0.5 μg of myc- HSPB5 (R120G) or (450 Δ A) or (464 Δ CT) mutants and either FRTTO as a control or V5 tagged HSPBs (HSPB1-10) at 1:3 ratio for 48 h. Cells were lysed with Triton X- 100 lysis buffer, fractionated into S (soluble) and P (pellet) fractions or T (total lysate) and western blotting was performed. The percentage of insoluble HSPB5 mutants in each case was quantified ([p]_density_*100 / ([p] _density_ + [s] _density_)) and is depicted in the chart above the blot. Values represent mean ± SD of 3 independent experiments.

In an attempt to elucidate how HSPB5 mutants cause protein aggregation and if and how this can be rescued, we co-expressed three different HSPB5 mutants (R120G, 450ΔA, 464ΔCT) with individual members of HSPB family (HSPB1-10) and tested their inhibitory effects on the aggregation-prone HSPB5 mutants in HEK293 cells. After co-expression for 48 h, cells were lysed with a 1% Triton X-100 lysis buffer and fractionated into soluble (S) and pellet (P) fractions. When expressed alone (FRTTO), the HSPB5 (R120G) mutant was nearly completely found in the pellet fraction (Fig 1). When co-expressed with the indicated HSPB members, HSPB1 and HSPB4 almost completely rescued R120G mutant from the detergent-insoluble pellet (Fig 1). HSPB5 and HSPB6 expression also substantially increased the solubilization of R120G whereas the other members were largely ineffective. Of note, levels of HSPB9 expression were too low to be conclusive. We found that neither HSPB1 nor HSPB4 had any effect on the total expression levels (T) of the R120G mutant (Fig 1 and S1 Fig).

We similarly found that the 450ΔA mutant, when singly expressed, translocated into the detergent insoluble fraction (Fig 1). When co-transfected with either HSPB1 or HSPB4, substantial solubilization was observed without prominent changes in the total expression levels of the mutant (Fig 1 and S1 Fig), albeit that the effects were more moderate than observed for the R120G mutant. Likewise, co-expression of wild type HSPB5 and HSPB6 separately had a minor effect as well.

For the 464ΔCT mutant we found again that HSPB1 and HSPB4 most strongly suppressed the insolubilization of 464ΔCT mutant and again HSPB5 and HSPB6 were also somewhat effective (Fig 1). Co-expression of HSPB1 and HSPB4 did reduce the total expression levels of the 464 ΔCT mutant by about 50% (Fig 1 and S1 Fig) unlike what we observed for the other 2 mutants.

To rule out any effect of the V5 tag on the functionality of the HSPBs, we also screened the effect of untagged versions on the aggregation-prone HSPB5 mutants. The results were comparable and confirmed that HSPB1 and HSPB4 were the most potent HSPB members for preventing the aggregation of all 3 HSPB5 mutants (S2 Fig).

Together, these data suggest a pattern in which HSPB1 and HSPB4 and to a lesser extent, HSPB6 and wildtype HSPB5 itself can counteract the aggregation of all three HSPB5 mutants. Strikingly, it was recently demonstrated that HSPB5 can form hetero-oligomeric complexes with exactly these three HSPB members [33,34]. Whilst in vitro, it can also co-oligomerise with HSPB8 [33], the member when expressed in cells, usuually does not form HSPB (hetero) oligomers but rather is present in a complex with Hsp70 and BAG3 [35]. This suggests that the reduction in the aggregation of HSPB5 mutants by those hetero-oligomeric partners is not mediated by the canonical chaperoning mechanisms such as refolding or degradation pathways.

**Fig 2:**
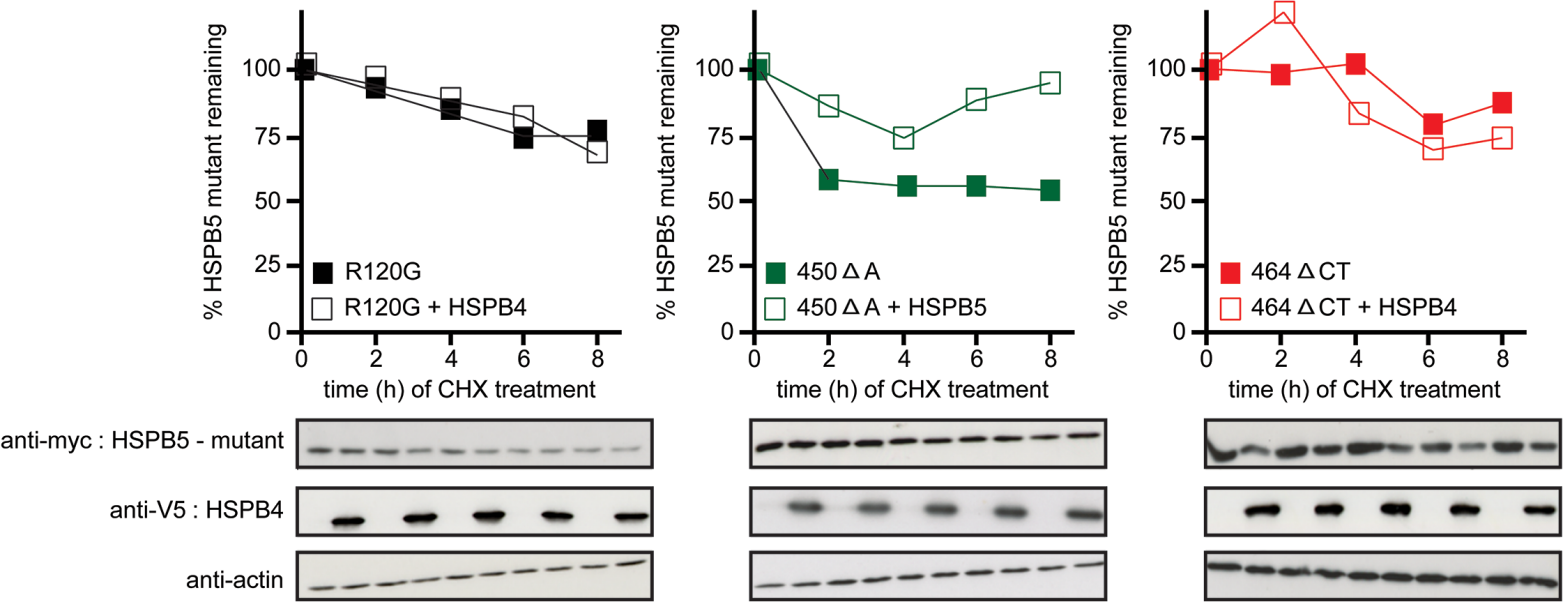
HSPB4 does not accelerate the degradation of HSPB5 mutants. HEK293 cells were co-transfected with myc-HSPB5 mutants (R120G, 450 Δ A, 464 Δ CT) and HSPB4-V5 or FRTTO as a control at 1:3 ratio. After 24 h, cells were treated with cycloheximide (50 μg/ml) for the indicated time points. Cells were lysed in SDS buffer and total lysates were collected. SDS-PAGE and western blotting were performed. The quantification of 3 independent experiments is depicted in the chart above each blot where each value was first normalized to its loading control then normalized to 0 time point.

Since, however, the steady state levels of the 464 ΔCT mutants were found to be lower when co-expressed with HSPB1 and HSPB4 (S1 Fig), we decided to directly investigate the possibility that the aggregation-rescuing members do so via accelerating the degradation rate of the HSPB5 mutants. Hereto, we performed cycloheximide chase experiments in cells co-expressing HSPB4 (used as the most effective example of the hetero-oligomeric partners) with the different HSPB5 mutants. Interestingly, we found that HSPB4 did not accelerate the degradation rate of the three studied HSPB5 mutants (Fig 2), implying that suppression of aggregation is not related to mutant protein degradation.

**Fig 3:**
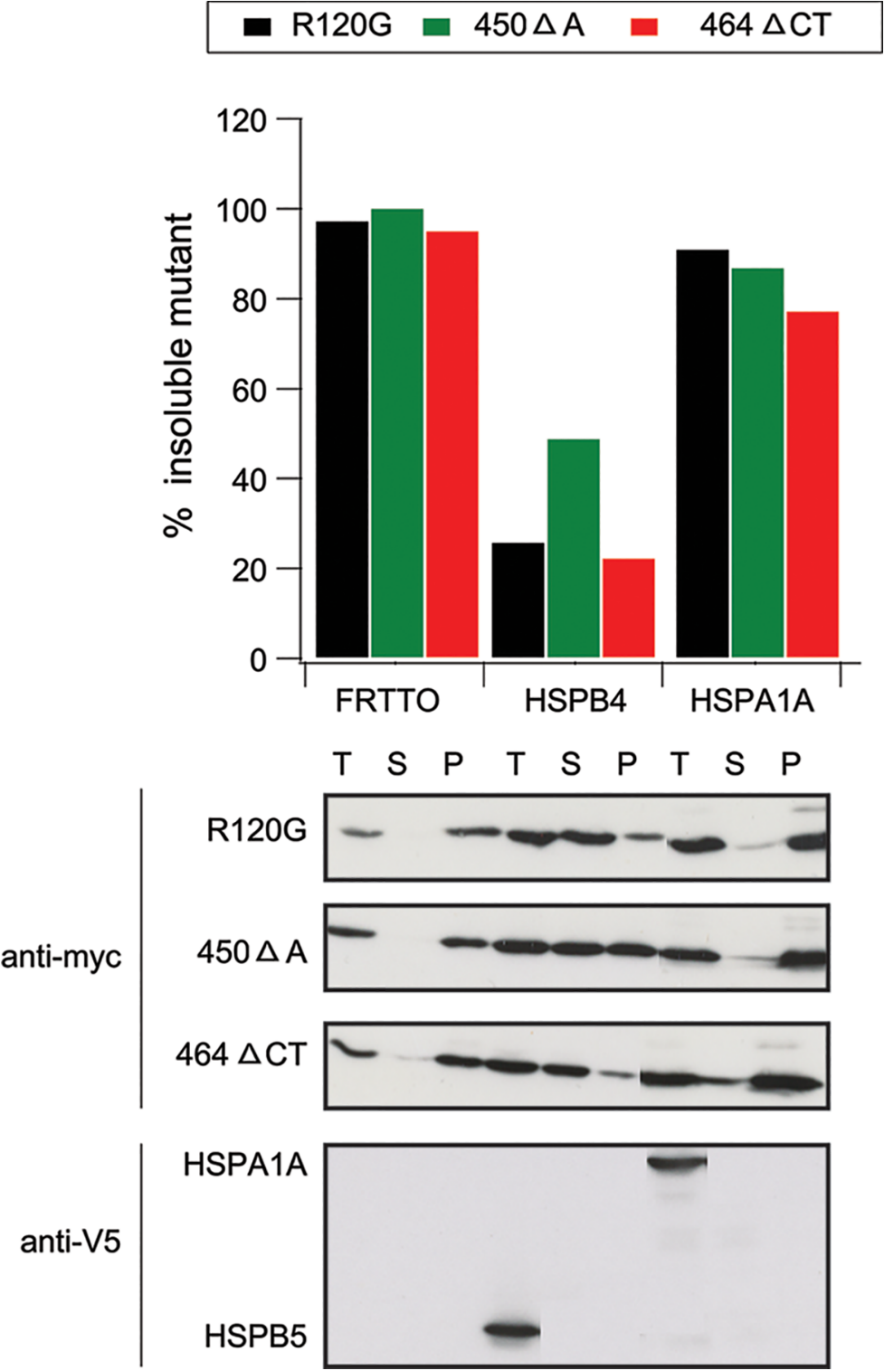
HSPA1A does not inhibit the aggregation of HSPB5 mutants. HEK293 cells were co-transfected with either one of the HSPB5 mutants (R120G, 450 Δ A, 464 Δ CT) and either an empty vector (FRTTO) or vectors encoding HSPB4 or HSPA1A at 1:3 ratio. 48 h after transfection, cells were lysed in Triton X 100 and fractionated into Soluble (S), Pellet (P) or total lysate (T). The percentage of insoluble HSPB5 mutants in each case was quantified ([p]_density_*100 / ([p] _density_ + [s] _density_)) and is depicted in the chart above the blot.

Given that the myopathies related to HSPB5 mutation are autosomal dominant and given that expression of the mutant alone is sufficient to cause aggregation, at least two possible scenarios could explain these phenomena. The first possibility is that the mutants cause aggregation via a dominant negative effect on the function of the hetero-oligomeric complexes leading to their aggregation (with clients). Alternatively, the mutants themselves are structurally instable and partially misfolded and thus are aggregation-prone and as such cause disease via a toxic gain-of-function. i.e the formation of aggregates *per se*. We first addressed the latter idea, by testing whether the insolubilization of the aggregation-prone HSPB5 mutants could be counteracted by overexpression of HSPA1A. HSPA1A itself can enhance refolding of heat aggregated proteins [36–38] and was shown to counteract the aggregation of several misfolded proteins upon overexpression in cells [39,40]. However, overexpression of HSPA1A did not reduce the aggregation of the HSPB5 mutants (Fig 3). This further indicates the aggregation of HSPB5 mutants is not via misfolding that requires handling by the HSP70 machinery and thus suggests that the rescue by the three HSPB members rather must be via counteracting a dominant-negative effect of the HSPB5 mutants on the functioning of HSPB hetero-oligomeric complexes. Dominant negative effects of the mutants may result from incorporation of a functionally dead component in such hetero-oligomeric complexes hereby leading to a secondary loss of function of the HSPB complexes that would be outcompeted if we co-express other functional components of the hetero-oligomeric complex as observed here.

We next decided to focus on the effect of HSPB4 on HSPB5 mutant-associated aggregation because of the fact that HSPB4 was the most potent inhibitor of mutant HSPB5 aggregation (together with HSPB1) and because HSPB4 (αA-crystallin) and HSPB5 (αB-crystallin) are closely related in their sequence and gene structure with 57% homology and they are often co-expressed in the lens [41]. This makes HSPB4 a biologically relevant candidate to deal with the deficits associated with HSPB5 mutations. Moreover, HSPB1 was previously shown to be a potent suppressor of the different HSPB5 mutants [25,29].

**Fig 4:**
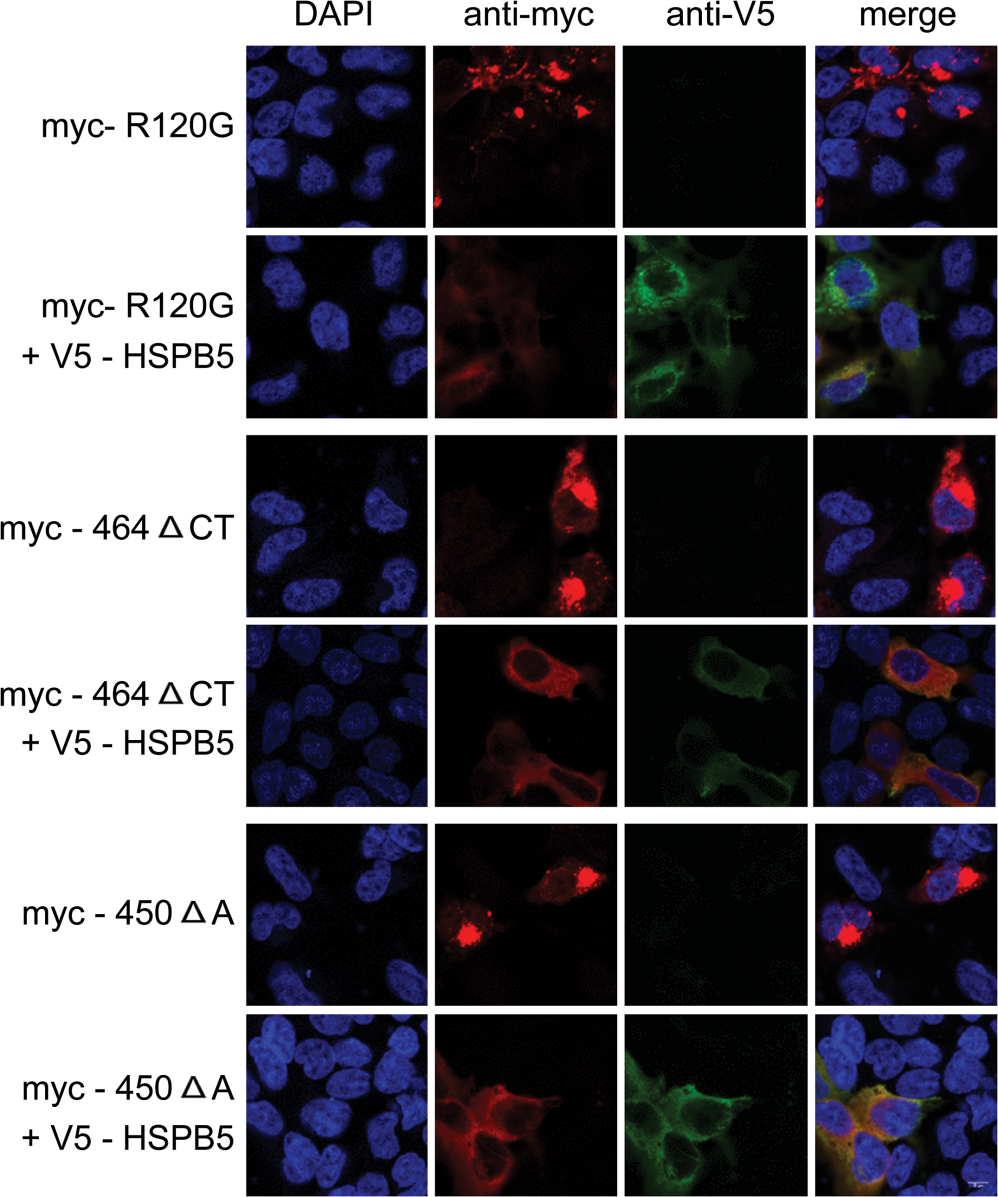
HSPB4 prevents the aggregation of HSPB5 mutants. Confocal images of transfected HEK293 cells with the myc-HSPB5 mutants: (R120G); top 2 rows, (464 Δ CT): middle 2 rows, or (450 Δ A): bottom 2 rows with or without co-expression of HSPB4-V5 at 1:3 ratio. 48 hours after transfection, cells were fixed and immunostained with mouse anti myc and rabbit anti V5 antibodies. DAPI was used to stain the nuclei.

First, we wished to confirm the biochemical analysis using confocal imaging. Hereto, HEK293 cells were transfected with the three different myc-tagged HSPB5 mutants and co-transfected without (FRTTO) or with HSPB4. When expressed alone, all HSPB5 mutants form dot-like structures suggestive of aggregates, which upon over expression of HSPB4 changed into a more diffuse signal (Fig 4) consistent with prevention of aggregation. Similar data were found for HSPB1, whilst HSPB7 was not effective (S3 Fig).

**Fig 5:**
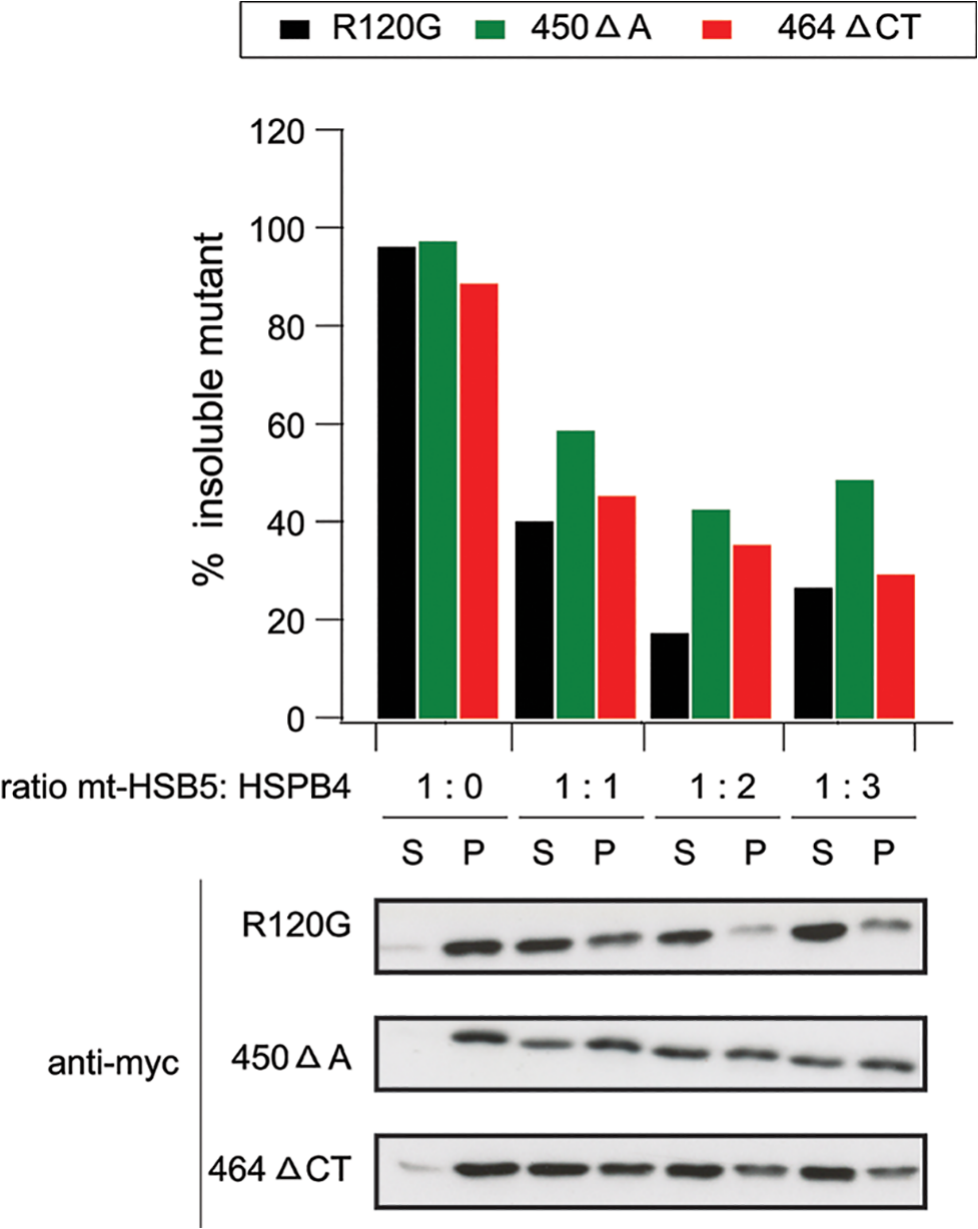
HSPB4 prevents the aggregation of HSPB5 mutants in C2C12 myoblasts. Mouse myoblast C2C12 cells were co-transfected with myc-HSPB5 mutants (R120G, 450 Δ A, 464 ΔCT) and FRTTO as a control (1:0 ratio) or HSPB4-V5 (at increasing ratios 1:1, 1:2, 1:3). 48 h after transfection, cells were lysed in Triton X-100 and fractionated into Soluble (S) and Pellet (P) and western blot was performed. The percentage of insoluble HSPB5 mutants in each case was quantified ([p]_density_*100 / ([p] _density_ + [s] _density_)) and is depicted in the chart above the blot.

Since HSPB5 mutants are associated with myopathies, we subsequently examined the ability of HSPB4 to prevent the aggregation of HSPB5 mutants in the mouse myoblast C2C12 cell line. Indeed, HSPB4 was also efficient in preventing the aggregation of the three studied HSPB5 mutants formed in C2C12 and in fact the effect was concentration dependent (Fig 5) and almost complete at HSPB4:HSPB5 ratios of 3:1, which interestingly is the same ratio at which the α-crystallin complex of the eye lens is most stable and shows the best chaperoning activity [42].

**Fig 6:**
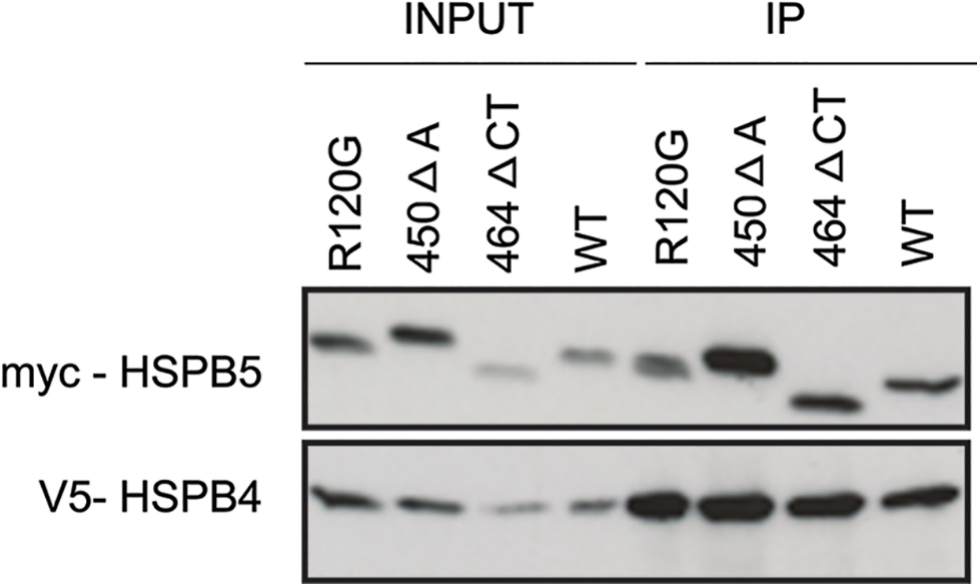
HSPB5 mutations do not disrupt the interaction with HSPB4. HEK293 cells were co-transfected with myc-tagged HSPB5 mutants (R120G, 450 Δ A or 464 Δ CT) and HSPB4-V5 at 1:3 ratio After 48 hours, cell lysates (Input) were immunoprecipitated with anti V5 (rabbit) antibody, followed by detection of HSPB5 mutants by an anti-myc antibody (IP).

To understand how HSPB4 may prevent mutant HSPB5 aggregation, we tested whether or not the different mutations on HSPB5 had affected the well-recognized interaction and complex formation between HSPB4 and HSPB5. Therefore, HEK293 cells were co-transfected with the 3 myc-tagged HSPB5 mutants and a V5-tagged HSPB4. 48 hours after transfection, cell lysates (input) were subjected to immunoprecipitation with an anti V5 antibody. Clearly, immunoprecipation for all 3 HSPB5 mutants was comparable to that of wild type HSPB5 (Fig 6) suggesting that none of the HSPB5 mutations affected the interaction with its partner, HSPB4.

## Discussion

In this study, we found that expression of HSPB5 mutants (R120G, 450 ΔA, 464 ΔCT) results in the formation of intracellular aggregates. Co-expression of HSPB1, HSPB4, HSPB5 (wild type) and HSPB6 but not the other HSPBs prevented the aggregation associated with all HSPB5 mutants. This was not only true in HEK293 cells, but also in mouse myoblast C2C12 cells, the cell types affected in myopathies cause by the HSPB5 mutants. Co-expression of HSPA1A (Hsp70) was ineffective in suppression the aggregation of the HSPB5 mutants.

Our data regarding HSPB1 are in line with most previous data showing that HSPB1 can prevent the aggregation associated with HSPB5 (R120G) mutant *in vitro* [25] as well as in cardiomyocytes [25], PTK2 cells [43] and Hela S3 cells [24]. In fact, in Hela S3 cells, HSPB1 prevented the aggregation of several different HSPB5 mutants including R11H, P20S, R56W, D109H, D140N, G154S, R157H and A171T [44]. In contrast, Zhang et al. 2010 found that in H9c2 cells, HSPB1 could not prevent the aggregation of the R120G and 450 ΔA HSPB5 but only that of 464 ΔCT mutant. Cell type dependent differences (e.g., related to the expression of the endogenous HSPB proteins) may, in part, underlie the different efficacy of HSPB1 to prevent mutant HSPB5 associated aggregation. The only 2 other HSPB family members so far tested on HSPB5 mutant aggregation are the wild type HSPB5, that could prevent HSPB5 (R120G) related aggregation in PTK2 cells [43], and HSPB8 that was found to be effective in vitro and in cardiomyocytes [25]. In fact, cardiac specific overexpression of HSPB8 in R120G transgenic mice reduced the aggregation and improved the heart function in vivo [26]. Other HSPBs were never systematically tested. Here, we find that besides HSPB1, HSPB5 (wild type), HSPB8, also HSPB6 and in particular HSPB4 can effectively reduce the aggregation of all three HSPB5 mutants tested.

Interestingly, the HSPBs that we find to be effective in preventing the aggregation of HSPB5 mutants were exactly those HSPBs that can form hetero oligomeric complexes with HSPB5 [33]. In line, Chavez Zobel 2003 showed that HSPB1 and wild type HSPB5 prevent the aggregation of HSPB5 (R120G) by co-oligomerizing with the mutant and we show that the most potent aggregation preventer, HSPB4, indeed immunoprecipitates with all three HSPB5 mutants. For the R120G and the 450ΔA mutant, aggregation suppression was not accompanied with lowered steady state levels and for the most potent suppressor, HSPB4, we showed it indeed was not associated with accelerated degradation. This suggests that, via the altered stoichiometry, these HSPB5 mutants may be stabilized in the hetero-oligomeric complexes. For the 464 ΔCT mutant, we do see about 50% lower levels as steady state, suggesting that in this case, the correcting chaperone do accelerate its degradation. This is in line with the findings of Zhang et al, who found that co-expression of HSPB1 with this HSPB5 mutant resulted in its accelerated proteasomal degradation [29]. Yet, for HSPB4, CHX chase experiments show that also for preventing the aggregation of this mutant, accelerated degradation seems to play no major role.

The HSPB members that rescue mutant HSPB5 associated aggregation also only partially overlap with those that (in the same HEK293 cells) e.g. can assist in refolding of heat denatured luciferase (HSPB1, HSPB4, HSPB5) [45] or that can prevent aggregation associated with the expression of proteins containing expanded polyglutamine stretches (HSPB6, HSPB7, HSPB8, HSPB9) [45] or mutant PARK2 (HSPB1, HSPB2, HSPB4, HSPB7, HSPB9) [46]. All these data, combined with the findings of a lack of an effect of HSPA1A on the aggregation of all three HSPB5 mutants and recent data from the group of Arrigo [47] that showing that the R120G mutants are not aggregation-prone, point towards the hypothesis that the rescue of the HSPB5 mutants by the different HSPB members is due to a stabilization of the hetero-oligomeric complexes, thus preventing a loss of its normal function when the stoichiometry of these mutants in these complexes are too highly altered.

## Supporting Information

S1 Fig Effect of the 10 human HSPB family members on the total levels of HSPB5 mutants. HEK293 cells were co-transfected with 0.5 μg of myc- HSPB5 (R120G) or (450 Δ A) or (464 Δ CT) mutants and either FRTTO as a control or V5 tagged HSPBs (HSPB1-10) at 1:3 ratio for 48 h. Cells were lysed with Triton X- 100 lysis buffer and total lysate (T) were collected. Western blotting was performed and the total HSPB5 mutant proteins were quantified relative to β-actin (loading control) and the data are plotted relative to the expression levels in control cells (FRTTO = 100%). Values represent mean ± SD of 3 independent experiments.
(TIF)

S2 Fig Effect of the 10 human untagged HSPB family members on the aggregation of HSPB5 mutants. HEK293 cells were co-transfected with either 0.5 μg of myc- HSPB5 (R120G) or (450 Δ A) or (464 Δ CT) and either FRTTO as a control or untagged HSPBs (HSPB1-10) at 1:3 ratio for 48 h. Cells were lysed with Triton X- 100 lysis buffer, fractionated into S (soluble) and P (pellet) fractions or T (total lysate) and western blotting was performed.
(TIF)

S3 Fig HSPB1, HSPB4 but not HSPB7 prevent the aggregation of HSPB5 mutants. Confocal images of transfected HEK293 cells with myc-HSPB5 mutants (R120G, 450 Δ A, 464 Δ CT) and either HSPB1-V5, HSPB4-V5 or HSPB7-V5 at 1:3 ratio. After 48 h, Cells were fixed and immunostained with mouse anti myc and rabbit anti V5 antibodies. DAPI was used to stain the nuclei. Scale bar ~10 μm.
(TIF)
