# Supplementary figures and images for: Rescue of αB Crystallin (HSPB5) Mutants Associated Protein Aggregation by Co-Expression of HSPB5 Partners
Source: PLoS One. 2015 May 11;10(5):e0126761. doi: 10.1371/journal.pone.0126761 (PMC4427338; doi:10.1371/journal.pone.0126761)

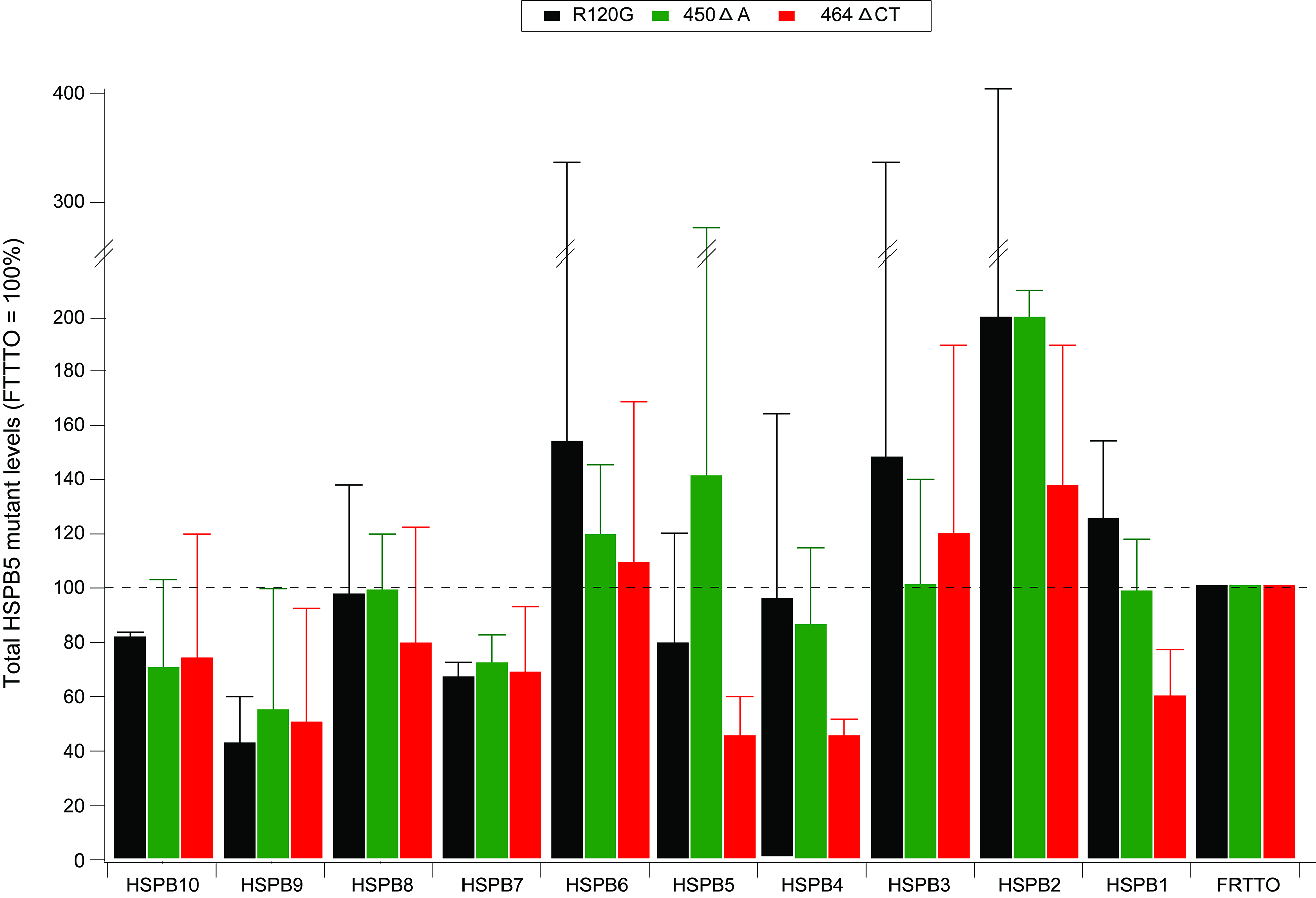

Supplement: S1 Fig — HEK293 cells were co-transfected with 0.5 μg of myc- HSPB5 (R120G) or (450 Δ A) or (464 Δ CT) mutants and either FRTTO as a control or V5 tagged HSPBs (HSPB1-10) at 1:3 ratio for 48 h. Cells were lysed with Triton X- 100 lysis buffer and total lysate (T) were collected. Western blotting was performed and the total HSPB5 mutant proteins were quantified relative to β-actin (loading control) and the data are plotted relative to the expression levels in control cells (FRTTO = 100%). Values represent mean ± SD of 3 independent experiments. (TIF) [file pone.0126761.s001.tif]

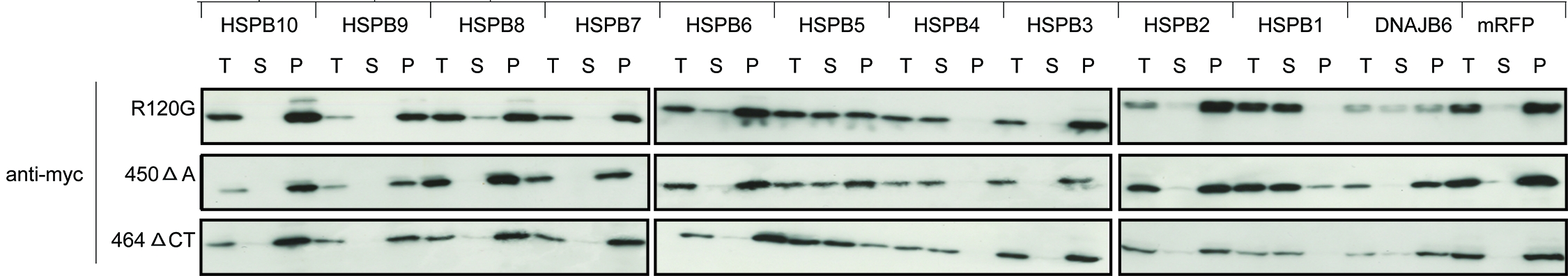

Supplement: S2 Fig — HEK293 cells were co-transfected with either 0.5 μg of myc- HSPB5 (R120G) or (450 Δ A) or (464 Δ CT) and either FRTTO as a control or untagged HSPBs (HSPB1-10) at 1:3 ratio for 48 h. Cells were lysed with Triton X- 100 lysis buffer, fractionated into S (soluble) and P (pellet) fractions or T (total lysate) and western blotting was performed. (TIF) [file pone.0126761.s002.tif]

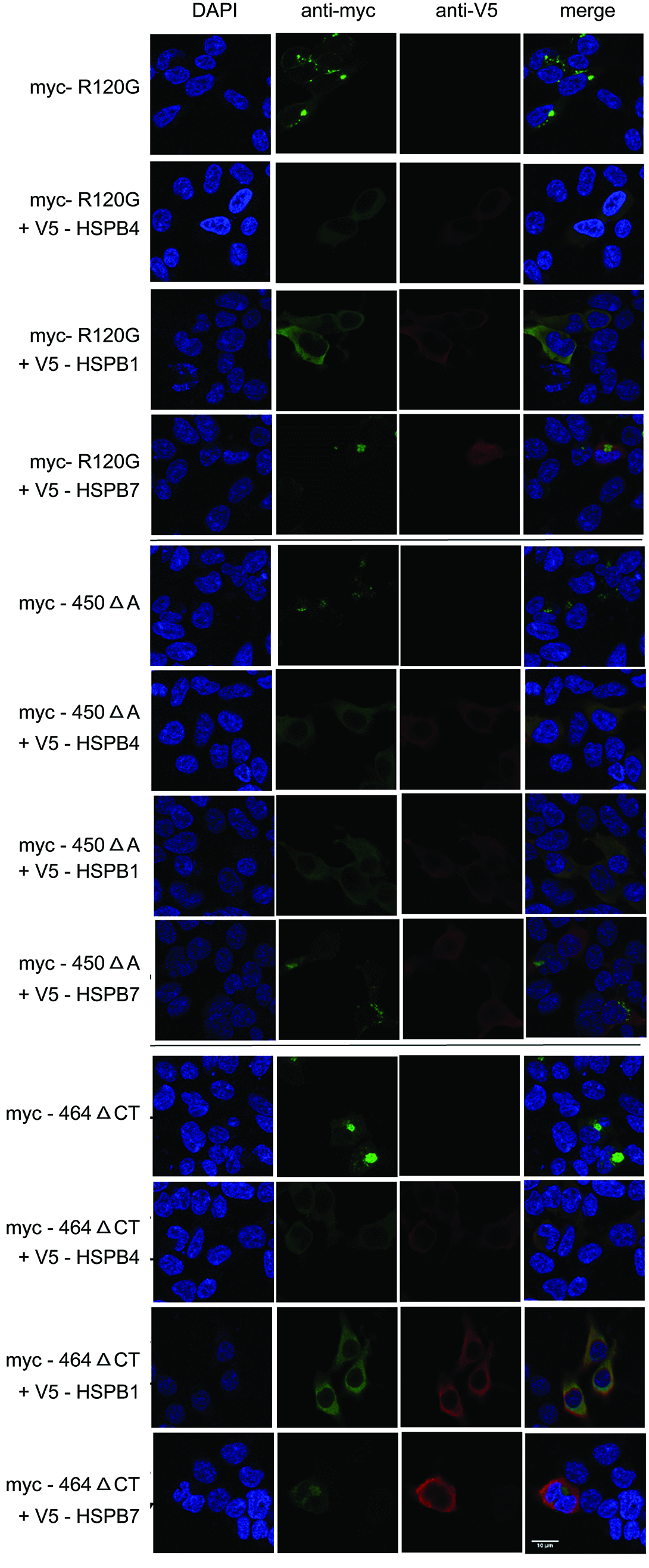

Supplement: S3 Fig — Confocal images of transfected HEK293 cells with myc-HSPB5 mutants (R120G, 450 Δ A, 464 Δ CT) and either HSPB1-V5, HSPB4-V5 or HSPB7-V5 at 1:3 ratio. After 48 h, Cells were fixed and immunostained with mouse anti myc and rabbit anti V5 antibodies. DAPI was used to stain the nuclei. Scale bar ~10 μm. (TIF) [file pone.0126761.s003.tif]
